# El profesional de la Medicina de Laboratorio ante la pandemia por COVID-19

**DOI:** 10.1515/almed-2020-0032

**Published:** 2020-05-18

**Authors:** Antonio Buño Soto

**Affiliations:** Servicio de Análisis Clínicos, Hospital Universitario La Paz, Paseo de la Castellana 261, Madrid 28046, Spain

**Keywords:** coronavirus, COVID-19, Medicina de Laboratorio

La actual pandemia declarada recientemente por la Organización Mundial de la Salud por la infección del nuevo coronavirus SARS2-CoV ha sido considerada como una alerta sanitaria que constituye una urgencia de salud pública de importancia internacional. La enfermedad comenzó a finales de diciembre pasado en Wuhan, provincia de Hubei en China y tras haber afectado de una manera muy importante tanto a China como a otros países de Asia, en estos momentos la propagación es de particular intensidad en Europa y Estados Unidos, habiendo sido diagnosticados pacientes en prácticamente todos los países del mundo [1].

En este siglo ya hemos asistido a distintos brotes de enfermedades causadas por virus que han supuesto un importante reto a nivel mundial. En 2002 se declaró un brote de neumonías por un nuevo patógeno, SARS Coronavirus. Años más tarde, en 2012 de nuevo otro Coronavirus causó un nuevo brote de infección respiratoria grave en Oriente Medio (MERS Coronavirus). En 2014 nos enfrentamos al mayor brote de enfermedad por el virus del Ébola que se originó en África Occidental. En algunos hospitales estos brotes motivaron la protocolización de la atención a pacientes con enfermedades de alta letalidad que en cierto modo ha sido aprovechado ahora.

Una irrupción tan rápida como la ocurrida en esta pandemia está cambiando rápidamente nuestras vidas de una forma inimaginable hasta hace bien poco y pone a prueba los sistemas sanitarios de todos los países afectados, entre ellos España. En estos momentos tan difíciles tenemos la obligación de ofrecer el mejor de los cuidados posibles a la población en general y a los pacientes en particular.

Como profesionales de la Medicina del Laboratorio no hemos sido ajenos a todo lo que está ocurriendo en nuestros hospitales y centros sanitarios. Desde el primer momento hemos sido pieza clave en la correcta organización de la asistencia. Desde el diagnóstico de la infección mediante la detección del virus en muestras de vías respiratorias hasta las pruebas necesarias para el correcto seguimiento, pronóstico y ayuda en la toma de decisiones terapéuticas, el laboratorio clínico está siendo una pieza absolutamente fundamental en el complicado puzle de esta nueva situación. Muchos compañeros viven esta experiencia con un espíritu de abnegada entrega con los pacientes, aunque en otras ocasiones se observan comportamientos más ilógicos quizás motivados por un temor irracional fruto del desconocimiento.

Nos hemos tenido que enfrentar en muy poco tiempo a numerosos y difíciles retos de los que pondremos algunos ejemplos:

– Hemos asistido a la paradoja de ver como nuestra actividad habitual casi se paralizaba mientras surgía una creciente demanda de atención a pacientes con la enfermedad por el nuevo coronavirus (COVID) hasta el punto de haberse convertido algunos hospitales en centros casi monográficos. Ello nos ha obligado a reajustar circuitos, protocolos y plantillas, asistiendo además a la merma de estas por el contagio de nuestros compañeros.
– También se nos ha pedido compaginar la reorganización de las tareas en nuestros servicios con ofrecer ayuda a otras ubicaciones del hospital para múltiples tareas. Incluso nos hemos visto abocados a organizar el laboratorio clínico de los “hospitales de campaña” que se han ido creando como apoyo a los ya existentes en tiempo récord y con enormes dificultades [2].
– Nos hemos tenido que revisar nuestros procedimientos de seguridad en el laboratorio y en algunos casos aprender a utilizar los equipos de protección individual. Nos han inundado de protocolos, recomendaciones y procedimientos que en muchos casos lejos de suponer una ayuda han contribuido a generar más confusión y, por, ende potencial caos entre nuestros compañeros.
– Ha habido que ejercitar nuestra capacidad de aprendizaje para formarnos en esta nueva entidad nosológica. En un tiempo récord han surgido numerosas publicaciones relacionadas con el nuevo virus (unas 1500 a fecha de 28 de marzo en Pubmed) de las que era necesario seleccionar las más relevantes y hacer lectura crítica de ellas para poder entender la enfermedad, ver que pruebas aportan mayor valor, que relevancia tiene medir d-dímero en este contexto, por qué nos demandan la interleucina 6 en estos pacientes, qué sentido tiene medir ferritina, marcadores de infección si/no, qué aportan los marcadores cardiacos, es útil o no trabajar con perfiles de diagnóstico, seguimiento ingreso en UCI, [3] … Todo ello sabiendo que nuestros compañeros clínicos esperan de nosotros cualquier ápice de ayuda que pueda aliviar su incertidumbre. Tengamos en cuenta que muchos de ellos han dejado por un tiempo de ser especialistas para pasar a ejercer de médicos con toda la humildad y el buen hacer del que son capaces.

Son momentos donde es necesario un liderazgo decidido desde las autoridades sanitarias hasta el último rincón de nuestros laboratorios. El entorno es muy cambiante y las decisiones se han de tomar de forma ágil, pero evitando caer en la precipitación. Es muy importante conservar la templanza suficiente en el atropellado día a día como para saber revisar la organización de nuestros servicios y laboratorios y adaptarla sobre la marcha en función de las circunstancias, en vez de caer en la tentación de ponemos en “modo hacer” porque la demanda lo requiere sin antes revisar si lo que hacemos tiene sentido. Saber tener la cabeza fría en estos momentos siempre suma.

Nuestros predecesores nunca nos contaron como enfrentarse a una situación así ni tampoco lo estudiamos en la universidad. Es momento de estar todos unidos, no solo como a nivel de la Medicina de Laboratorio sino con todos los profesionales de nuestros hospitales y centros de trabajo. Pongamos todo nuestro mejor saber hacer en marcha y confío que cuando pase esta inusual situación sepamos reflexionar para aprender de nuestros errores. Será la mejor manera de estar bien preparados para la siguiente.
